# Supplementary material for: Physiological and Biochemical Responses of Pseudocereals with C3 and C4 Photosynthetic Metabolism in an Environment with Elevated CO2
Source: Plants (Basel). 2024 Dec 9;13(23):3453. doi: 10.3390/plants13233453 (PMC11644615; doi:10.3390/plants13233453)
Supplement: Supplementary file 1 [file plants-13-03453-s001.zip › plants-3320824-supplementary.pdf]

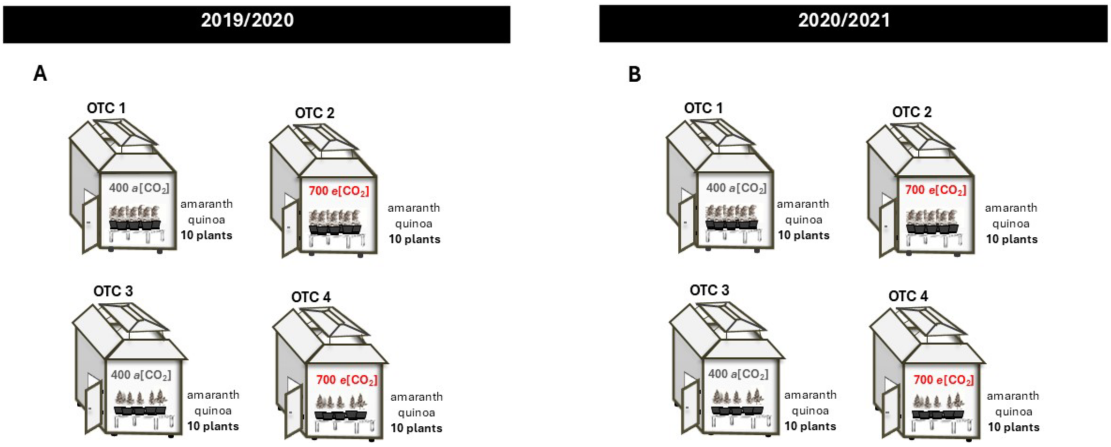

Supplementary figure 1. Experimental setup to $a[CO_2]$ versus $e[CO_2]$ studies in amaranth and quinoa plants to two agricultural years, 2019/2020 (A) and 2020/2021(B).

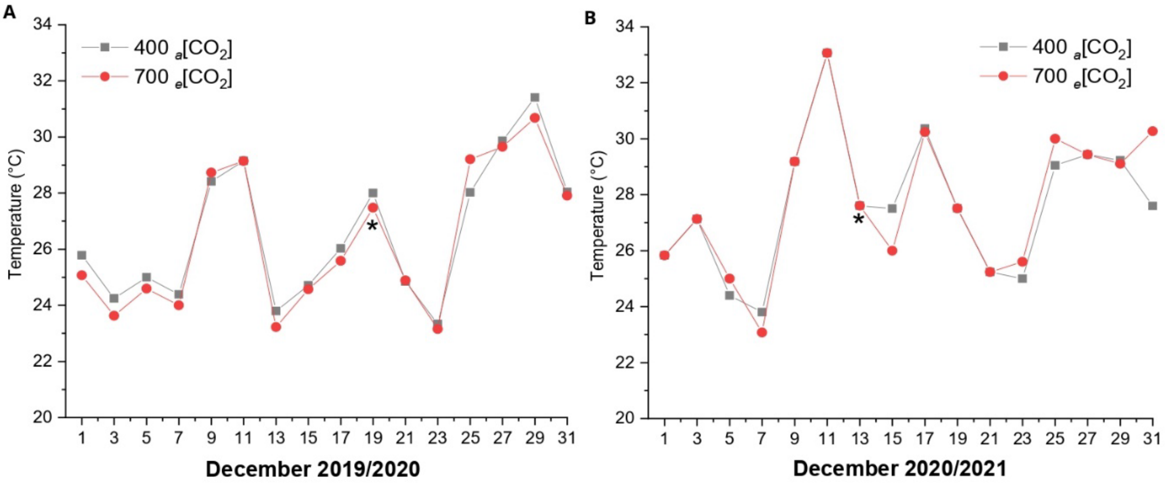

Supplementary figure 2. Daily mean temperature inside the OTCs during 2019/2020 (A), and 2020/2021 (B). *Transition period between vegetative and flowering .
